# A Model-Based Method for Estimating the Attitude of Underground Articulated Vehicles

**DOI:** 10.3390/s19235245

**Published:** 2019-11-28

**Authors:** Lulu Gao, Fei Ma, Chun Jin

**Affiliations:** School of Mechanical Engineering, University of Science & Technology Beijing, Beijing 100083, Chinajinjinbit@163.com (C.J.)

**Keywords:** underground articulated vehicles, attitude estimation, IMU, kinematic model, Kalman Filter

## Abstract

This paper presents a novel model-based method for estimating the attitude of underground articulated vehicles (UAV). We selected the Load–Haul–Dump (LHD) vehicle as our application object, as it is a typical UAV. First, we established the involved models of the LHD vehicle, including a kinematic model, the linear and angular constraints of a center articulation model, and a dynamic four degrees-of-freedom (DOF) yaw model. Second, we designed a Kalman filter (KF) to integrate the kinematic and constraint models with the data from an inertial measurement unit (IMU), overcoming gyroscope drift and disturbances in external acceleration. In addition, we designed another KF to estimate the yaw based on the dynamic yaw model. The accuracy of the estimations was further enhanced by data fusion. Then, the proposed method was validated by a simulation and a field test under different dynamic conditions. The errors in the estimation of roll, pitch, and yaw were 3.8%, 2.4%, and 4.2%, respectively, in the field test. The estimated longitudinal acceleration was used to obtain the velocity of the LHD vehicle; the error was found to be 1.2%. A comparison of these results to those of other methods showed that the proposed method has high precision. The proposed model-based method will greatly benefit the location, navigation, and control of UAVs without any artificial infrastructure in a global positioning system (GPS)-free environment.

## 1. Introduction

With the increase in depth of underground mines, a hazardous environment and safety problems are becoming increasingly prominent. Autonomous vehicles (AVs) are urgently needed to ensure that underground mines are safe and efficient [1,2]. As a typical and important kind of underground vehicle, underground articulated vehicles (UAVs) are widely used in underground mines due to their advantages of a higher maneuverability and efficiency [3]. Therefore, automated UAVs (AUAVs) have first priority in underground mines. However, the automation of their precise control and navigation requires related information on the target vehicle [4,5,6], which includes the attitude, velocities, and even accelerations relative to different directions [7].

**Figure 1:**
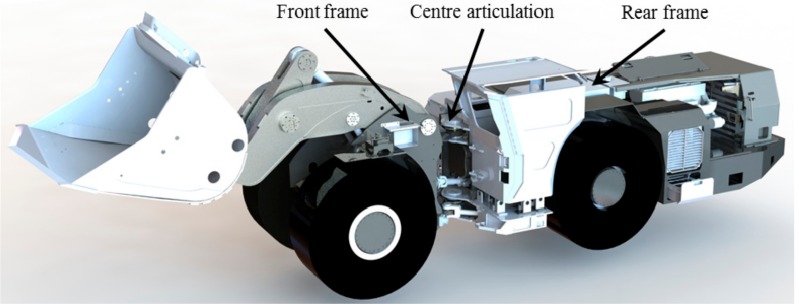
The typical structure of a Load–Haul–Dump (LHD) vehicle.

The acquisition of the attitude and states of vehicles has been studied intensively for many years [8,9,10,11]. What needs to be emphasized is that, although a global positioning system (GPS) can provide the related information with high accuracy, obstruction of the signal can cause the system to be invalid in some urban areas and tunnels, which is an especially serious problem in underground mines [9]. Two kinds of methods were designed in order to cope with this issue with UAVs according to the specific characteristics of underground mines: infrastructure-based methods [8] and sensor-based methods [12,13]. In addition, a mixture of both is sometimes used [14]. As efficient vehicles for underground mines, Load–Haul–Dump (LHD) vehicles, as shown in Figure 1, are considered to be study and application objects.

Infrastructure-based methods were applied during an early stage, but have not been widely used due to their cost and obstructions in the environment [8,15].

Regarding the sensor-based methods, sensors with various functions, such as laser scanners [16], odometers [9,17], and charge-coupled device (CCD) cameras [18] have been applied to obtain the states of vehicles in underground mines. Mäkelä [17] designed a guidance system for LHD vehicles that required no extra infrastructure in the tunnel, and where only the yaw motion was considered. The yaw angle of the front and rear frames was obtained by a gyroscope, an angle encoder, and an odometer; however, the accuracy of the yaw angle was not given. Chi et al. [16] designed a positioning system based on laser sensors, in which the yaw angle is measured and corrected based on barcode theory, and the authors only provided the longitudinal error from a field test, which was less than 15 cm after 40 m of travel. For these sensor-based methods, the involved sensors need external signals; for example, the lasers need to receive reflected signals, and the cameras take pictures based on the reflected light. However, the dusty and dim environment of an underground mine can obstruct these signals [19], which can bring about uncertainties and errors during the process of measurement. For these reasons, the robustness of these methods will be poor. Additionally, the data from the gyroscope provides basic information on the yaw motion, gyroscope drift is not considered, and the error that accumulates can only be corrected with the assistance of beacons. Sometimes, continuous estimation during operation cannot be realized, which can seriously restrict the speed and efficiency [20].

To overcome these difficulties with the infrastructure-based and sensor-based methods, some special devices and algorithms have been applied. Marshall et al. [21] designed a system without infrastructure for the automation of an LHD vehicle to improve the robustness and reliability. Laser rangefinders, a wheel odometer, and angle encoders were applied to provide the necessary data. An extended Kalman filter (EKF) was used to estimate the planar states, which included position and orientation. Although the errors in the estimation were not provided, the path-tracking errors in the heading angle and the lateral angle, which were less than 0.1 rad and 0.28 m, respectively, demonstrated the validity of the system. Wu et al. [22] presented a system for estimating the yaw motion of an LHD vehicle, in which two ultra-wide band (UWB) modules were adopted and planar motion was assumed for the LHD vehicle. A field test showed that the error in the estimation was less than 3°. Jin et al. [9] proposed a model-based method for estimating the states of electric drive UAVs, in which an unscented Kalman filter (UKF) and a dynamic model were used. The estimated error in the longitudinal velocity, yaw motion, and side-slip was less than 5% under pavement conditions, which was a cement pavement. Although the accuracy was found to be acceptable, the uncertainties in the parameters in the dynamic model may bring about errors under uneven road conditions. Byeolteo et al. [23] presented a novel method for obtaining the states of position and direction with a magnetic field and simultaneous localization and mapping (SLAM) in an underground environment. Regarding the position and direction, results from field tests showed that the proposed method reduced the root mean square (RMS) of the errors by 95.56% and 99.34%, respectively, compared with the results of dead reckoning. Although the robustness of the system was enhanced, some basic installations were necessary, and most of the sensors required a feasible environment. Moreover, the method was not verified on mine-like roads, where the road is terrible and bumpy.

The gyroscope that was used in the abovementioned methods has no special requirements for operation in a mine environment, which makes it the preferred sensor. However, a high-grade gyroscope is too expensive to be widely used, and the drift in a general gyroscope may also lead to errors in the results. With the advances in micro-electro-mechanical systems (MEMS), inertial measurement units (IMUs), in which a gyroscope and an accelerometer are included, have been widely used for the estimation of attitude and positioning. Zhu et al. [24] presented a novel system for estimating the attitude and measuring the stability of articulated heavy vehicles. A set of IMU sensors formed the hardware foundation of the system, and several sensors were adopted by considering the special structure of articulated vehicles. The states of the target vehicle were obtained by data fusion. A field test showed that the errors in the pitch, roll, and yaw angles in a static state were 0.027°, 0.025°, and 0.426°, respectively. The pitch and roll motions were found to vary between ±20°, and the yaw motion was found to vary between 0° and 60°. The corresponding errors were all less than 4%. However, the results were obtained on pavement, and the system did not cope well with gyroscope drift.

Drift in the gyroscope and interference with the accelerometer remain key problems with IMUs, and some methods have been proposed to improve the accuracy of estimations. These include the threshold-based switching approach [25], the adaptive filter method [26], a model-based algorithm [10], and a comprehensive algorithm [27]. Furthermore, Ahmed et al. [28] investigated a novel algorithm for attitude estimation using low-cost IMU sensors, which was applied to a land vehicle. A kinematic model and the vector norm property of gravity were used to estimate the pitch and roll angles. After that, the obtained results were used to estimate the yaw angle in combination with a magnetometer. The results of a simulation and field tests showed that the proposed algorithm was robust and accurate under different dynamic conditions.

Most of the target vehicles that were studied in previous papers were four-wheeled commercial vehicles [29], which can be considered to be single rigid bodies. However, different from the target vehicles in the abovementioned studies, UAVs contain two parts, which are connected to each other with center articulation [24]. Although some methods have been applied to articulated vehicles, the dynamics, the kinematic model, and the defects in IMU sensors have not been considered. Moreover, the roll and pitch angles of UAVs have been neglected in related research [9,22], as the motion of the target vehicle was always considered to be on a plane. The motions of UAVs involve violent pitch and roll motions that occur during normal operation between different levels of underground mines or on an uneven road. Estimations of roll and pitch are essential to the navigation and dynamical control of UAVs in an underground mine environment.

In addition, as the most important piece of information about a target vehicle, the yaw motion is measured by a magnetometer in an IMU. The magnetometer measures the magnetic force between different yaw motions to obtain the yaw angle. Unfortunately, UAVs are usually put to work in an underground metal mine, where the magnetic field may be disordered by metallic minerals. In addition, in the commonly used method, the accuracy of the yaw angle’s estimation cannot be improved by using data on acceleration. Therefore, the yaw angle of a UAV cannot be obtained by an IMU alone. Inspired by the work in [9], we use the information from measurements and a kinematic model of center articulation to improve the accuracy of yaw angle estimation, which is obtained primarily by a dynamic model. Motivated by the above discussion, we make a slight improvement in the accuracy of the estimation of the state of a UAV. The three original contributions that distinguish the present work from previous works can be summarized as follows:

(1) A novel model-based method for estimating the attitudes of UAVs is proposed. In the first step, the kinematic model and the constraint of center articulation models are used to overcome the drift in the IMU.
(2) A model-based algorithm that combines the dynamic model with a KF is developed to estimate the yaw motion of an LHD vehicle.
(3) A fusion of the data from different models and sensors is carried out to improve the accuracy of attitude estimations.

The remainder of this paper is organized as follows. In Section 2, the involved models are established, these models are embedded into the KFs, and algorithms are described for the pitch and roll motion and yaw motion. In Section 3, a simulation of an LHD vehicle is carried out under different dynamic conditions, and data from the simulation are used to verify the method. Section 4 presents the results of a field test and a discussion. Finally, Section 5 summarizes our conclusions and future work.

## 2. Method

**Figure 2:**
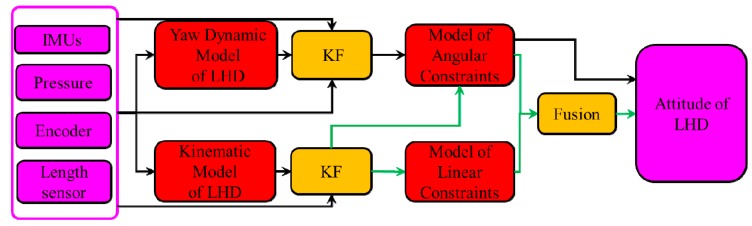
A block diagram of the model-aided method for estimating the attitude of an LHD vehicle.

The proposed method is briefly described in the diagram shown in Figure 2. Some sensors are necessary, including IMUs, an encoder, a pressure sensor, and a length sensor. In Figure 2, the green line denotes the attitude of the roll and the pitch. KFs are the major algorithms. The yaw and other attitudes are obtained separately. In Figure 2, the black line after the angular model denotes the yaw of the LHD vehicle, and the green line denotes the other attitudes. First, the data from the IMU that is located on the front frame are used to obtain the dynamic acceleration and attitude (roll and pitch) of the front frame. During this process, the kinematic model of the front frame and the norm characteristics of the gravitational acceleration vector are used by the KF. Second, the information on acceleration is combined with linear constraints to calculate the acceleration of the rear frame, which can be used to calculate the attitude of the pitch and roll of the rear frame. Simultaneously, the attitude of the front frame is used to calculate the attitude of the rear frame with angular constraints. Additionally, an improvement is carried out with data fusion to obtain the final attitude of the LHD vehicle. The dynamic model of yaw is used to obtain the yaw of the front frame with the help of the KF and data from the sensors. The corresponding part of the rear frame is obtained by the model of angular constraints.

### 2.1. Models

#### 2.1.1. Kinematic Model of the LHD Vehicle

**Figure 3:**
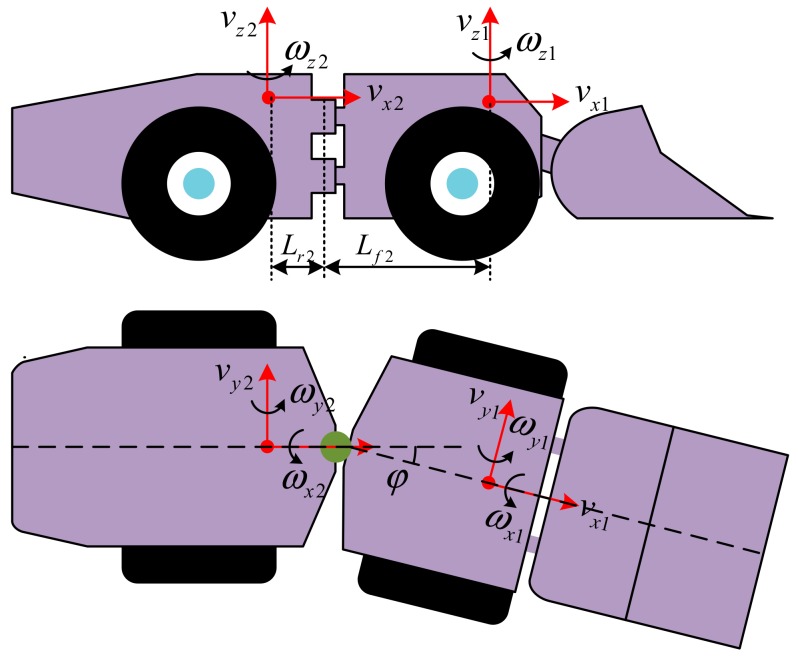
Directions of the linear and angular velocities of the LHD vehicle.

The directions of the linear and angular velocities of the LHD vehicle are defined as shown in Figure 3. The front and rear frames are considered to be rigid bodies. According to the motion of a rigid body, the kinematic model of the front frame and the rear frame of the LHD vehicle can be expressed as:

(1a) $$a_{x1}={\dot{v}}_{x1}-\omega_{z1}v_{y1}+\omega_{y1}v_{z1}$$

(1b) $$a_{y1}={\dot{v}}_{y1}+\omega_{z1}v_{x1}-\omega_{x1}v_{z1}$$

(1c) $$a_{z1}={\dot{v}}_{z1}-\omega_{y1}v_{x1}+\omega_{x1}v_{y1}$$

(1d) $$a_{x2}={\dot{v}}_{x2}-\omega_{z2}v_{y2}+\omega_{y2}v_{z2}$$

(1e) $$a_{y2}={\dot{v}}_{y2}+\omega_{z2}v_{x2}-\omega_{x2}v_{z2}$$

(1f) $$a_{z2}={\dot{v}}_{z2}-\omega_{y2}v_{x2}+\omega_{x2}v_{y2}$$

where the subscripts *x*, *y*, and *z* indicate the different directions in Figure 3. The subscripts 1 and 2 indicate the front frame and the rear frame of the LHD vehicle, respectively. 

The kinematic constraints were obtained between the two frames, which contain linear and angular constraints. More specifically, the linear constraints in different directions can be expressed as:

(2a) $$v_{x2}=v_{x1}\cos\varphi -\left(v_{y1}-L_{f2}\omega_{z1}\right)\sin\varphi$$

(2b) $$v_{y2}=v_{x1}\sin\varphi -\left(v_{y1}-L_{f2}\omega_{z1}\right)\cos\varphi -L_{r2}\omega_{z2}$$

(2c) $$v_{z2}=v_{z1}+\left(L_{f2}+L_{r2}\right)\omega_{y1}$$

where *φ* is the steering angle of center articulation, and *L_f_*_2_ and *L_r_*_2_ are the distance from the center of gravity of the front frame and the rear frame to the point of articulation, respectively, as shown in Figure 3. Similarly, the angular constraints in different directions can be expressed as:

(3a) $$\theta_{2}=\theta_{1}\cos\varphi +\phi_{1}\sin\varphi$$

(3b) $$\phi_{2}=\phi_{1}\cos\varphi +\theta_{1}\sin\varphi$$

(3c) $$\varphi =\psi_{1}-\psi_{2}$$

where *θ*, *φ*, and *Ψ* are the pitch, roll, and yaw angle, respectively.

According to [10], the external acceleration must be removed to improve the accuracy of pitch and roll estimation. The constraints (2a)–(2c) may be useful for the removal of external acceleration. In addition, Equations (3a)–(3c) can also be used to estimate the attitude of the rear frame. However, neither of them can be the only source of the attitude’s estimation because of the overly ideal assumptions and the errors in the measurement.

#### 2.1.2. Dynamic Model of Yaw for the LHD Vehicle

**Figure 4:**
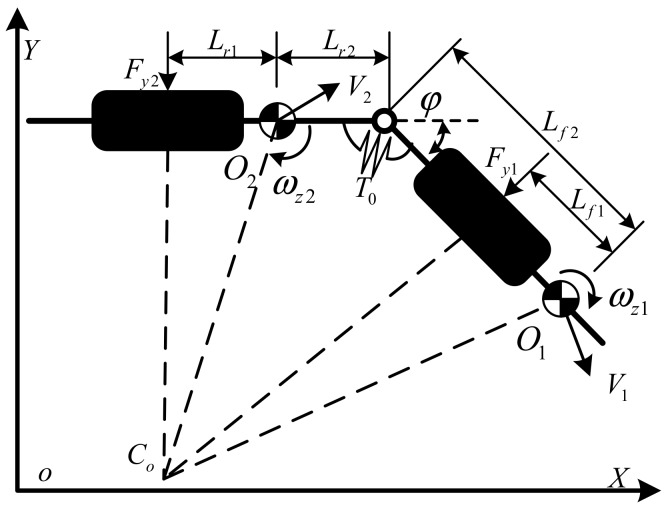
A dynamic model of yaw for the LHD vehicle.

The dynamic model of yaw is used to restrain the drift in the yaw information from the gyroscope in the IMU. Considering the efficiency of the method, a simplified dynamic model of the LHD vehicle was adopted, which is shown in Figure 4. The directions of the motions are the same as the directions that are defined in Figure 3.

According to Newton’s second law, the simplified dynamic model can be given by:

(4a) $$\left(I_{z1}+m_{1}L_{f2}^{2}\right){\dot{\omega }}_{z1}=T_{0}-F_{y1}\left(L_{f2}-L_{f1}\right)$$

(4b) $$\left(I_{z2}+m_{2}L_{fr}^{2}\right){\dot{\omega }}_{z2}=-T_{0}+F_{y2}\left(L_{r2}+L_{r1}\right)$$

(4c) $$m_{1}{\dot{v}}_{x1}+m_{2}{\dot{v}}_{x2}\cos\varphi =F_{xf}+F_{xr}\cos\varphi -F_{yr}\sin\varphi$$

(4d) $$m_{1}{\dot{v}}_{y1}+m_{2}{\dot{v}}_{y2}\cos\varphi =F_{yf}+F_{yr}\cos\varphi -F_{xr}\sin\varphi$$

where *I*_z1_ and *I*_z2_ are the moment of inertia of the front frame and the rear frame, respectively. *F_xf_*, *F_xr_*, *F_yf_*, and *F_yr_* are the force of front and rear tire in X and Y directions, respectively. *T*_0_ is the steering moment, and although it can be modeled as an equation of *ϕ* in a steady state [9], it normally operates in an unsteady state. Here, we consider the steering moment as the input of the dynamic model, and it can be indirectly measured by the pressure and displacement sensors [30]. It is sufficient to focus on one of the two yaw motions in Equation (4) considering the constraints in Equation (3c).

In addition, the cornering force of a tire can be given by:

(5) $$F_{yi}=k_{i}\alpha_{i}$$

where *k_i_* and *α_i_* are the side-slip stiffness and the angle of the tire, respectively. They can be described by:

(6a) $$\alpha_{1}=\left(v_{y1}-L_{f1}\omega_{1}\right)/v_{x1}$$

(6b) $$\alpha_{2}=\left(v_{y1}+\left(L_{r1}-L_{f2}-L_{r2}\right)\omega_{z1}+\left(L_{r2}-L_{r1}\right)\dot{\varphi }\right)/v_{x1}+\varphi \;.$$

Therefore, the state equation of the dynamic model of yaw for the front frame can be given by:

(7) $${\dot{\omega }}_{z1}=T_{0}/\left(I_{z1}+m_{1}L_{f2}^{2}\right)-k_{1}\beta \left(L_{f2}-L_{f1}\right)/\left(I_{z1}+m_{1}L_{f2}^{2}\right)+k_{1}\omega_{z1}L_{f1}\left(L_{f2}-L_{f1}\right)/\left(\left(I_{z1}+m_{1}L_{f2}^{2}\right)v_{x1}\right)$$

where the sideslip angle *β* can be expressed as:

(8a) $$\beta \approx v_{y1}/v_{x1}\approx L_{f1}/R_{1}$$

(8b) $$R_{1}=\left(L_{f2}\cos\varphi +L_{r2}\right)/\sin\varphi \;.$$

In addition to the kinematic model and the dynamic model, a model of the sensors should also be taken into account.

#### 2.1.3. Model of the Sensors

Two kinds of sensor are applied in the proposed method, i.e., an IMU sensor and an angular encoder. Considering the noise in the signals from the involved sensors, which include a tri-axis gyroscope, a tri-axis accelerometer, and an angular encoder, the models of the involved sensors can be described in the corresponding frame as follows:

(9a) $$y_{i,G}=\omega_{i,G}+n_{i,G}$$

(9b) $$y_{i,A}=g_{i,A}+a_{i,A}+n_{i,A}$$

(9c) $$y_{v}=v_{x}+n_{v}$$

(9d) $$y_{c}=\gamma_{c}+n_{c}$$

where the subscript *i* = 1,2 denotes the front frame and the rear frame of the LHD vehicle, respectively, ***y****_i_***_,G_** is a column vector [*ω_i_*_,Gx_ *ω_i_*_,Gy_ *ω_i_*_,Gz_]^T^, which contains three angular velocities of the X, Y, and Z axes in the frame of installation, vector ***ω****_i_*_,***G***_ is the corresponding true value [*ω_i_*_,x_ *ω_i_*_,y_ *ω_i_*_,z_]^T^, and ***n****_i_*_,***G***_ is white Gaussian noise with a zero mean about each axis. In Formula (9b), ***y****_i_***_,A_** is also a column vector [*g_i_*_,Ax_ *g_i_*_,Ay_ *g_i_*_,Az_]^T^, which contains the acceleration of gravity ***g****_i,**A**_* **=** [*g_i_*_,x_ *g_i_*_,y_ *g_i_*_,z_]^T^, the true acceleration of the dynamics of the target ***a_A_* =** [*a_i_*_,Ax_ *a_i_*_,Ay_ *a_i_*_,Az_]^T^, and the corresponding white Gaussian noise with a zero mean ***n****_i_*_,A_. Similarly, in Formula (9c), *y_v_* is a scalar value of the longitudinal velocity from the angular encoder including the true value *v_x_* and noise *n_v_*. In Formula (9d), y*_c_* is also a scalar value of the angular transducer, which contains the true angle *ϕ*_c_ and noise *n*_c_.

### 2.2. State Vector

#### 2.2.1. State Vector of Pitch and Roll

Considering the conditions of an LHD vehicle, the Euler angle method is a good choice for attitude representation. Then, the states of the LHD vehicle can be defined as pitch (*θ_i_*), roll (*ϕ_i_*), and yaw (*ψ_i_*). The transformation relationship between the vehicle coordinate frame (VX) and the navigation coordinate frame (NX) can be expressed as [27]:

(10) $$NX{}_{i}=T_{i}VX{}_{i}$$

where ***T****_i_* is the transformation matrix, and the subscripts *i* = 1,2 represent the front frame and the rear frame, respectively. The transformation matrix can be expressed as:

(11) $$T_{i}=\left[\begin{array}{ccc} c\psi_{i}c\theta_{i} & c\psi_{i}s\theta_{i}s\phi_{i}-s\psi_{i}c\phi_{i} & c\psi_{i}s\theta_{i}c\phi_{i}+s\psi_{i}s\phi_{i}\\ s\psi_{i}c\theta_{i} & s\psi_{i}s\theta_{i}s\phi_{i}-c\psi_{i}c\phi_{i} & s\psi_{i}s\theta_{i}c\phi_{i}-c\psi_{i}s\phi_{i}\\ -s\theta_{i} & c\theta_{i}s\phi_{i} & c\theta_{i}c\phi_{i} \end{array}\right]$$

where *c* and *s* are the trigonometric functions cosine and sine, respectively. We note that the last row of the transformation matrix only contains the pitch and roll angles, which can be used to calculate these angles with:

(12a) $$\phi_{i}=\arctan(T_{i32}/T_{i33})$$

(12b) $$\theta_{i}=\arctan(-T_{i31}/(T_{i33}/sin\phi_{i}))$$

where ***T****_i_*_*j k*_ is the (*j*, *k*)-th element of ***T****_i_*.

As mentioned in [10], the state vector of a target vehicle at time *t*, which is also actual state, can be expressed as:

(13) $$X_{i,\;t}={\left[\begin{array}{ccc} T_{i31} & T_{i32} & T_{i33} \end{array}\right]}^{T}.$$

The acceleration of gravity in Equation (9b) can be given by:

(14) $$g_{i,A}=gX_{i,\;t}$$

where *g* is the gravitational acceleration constant, which is 9.81 m/s^2^.

#### 2.2.2. State Vector of Yaw

According to Equation (7), the yaw motion of the front frame can be estimated by a state equation, and the corresponding angle of the rear frame can be obtained by Equation (3c). Therefore, the state vector of yaw motion can be defined as:

(15) $$X_{z,\;t}=\omega_{z1}.$$

### 2.3. Design of KFs

#### 2.3.1. KF for Pitch and Roll

The model of a linear KF, which includes the model of process and measurement, can be expressed as:

(16a) $$X_{i,t}=\Phi_{i,t-1}X_{i,t-1}+w_{i,t-1}$$

(16b) $$z_{i,t}=H_{i}X_{i,t}+v_{i,t}$$

where ***X****_i_*_,*t*_ is the state vector at time *t* as defined in Equation (13), ***Φ****_t_*_−1_ is the state transition matrix, ***H****_i_*_,*t*_ is the observation matrix, and ***w*** and ***v*** are the white Gaussian noise in the process and measurement, respectively.

The transformation matrix can be calculated with an integration from *t* − 1 to *t* [13]:

(17) $$T_{i,t}=T_{i,t-1}\left(I_{3}+\Delta t{\tilde{\omega }}_{i,t-1}\right)$$

where ***I***_3_ is a 3 × 3 identity matrix, Δ*t* is the interval of the sampling, and the symbol―denotes a cross-product of the target vector [27].

Not all of the terms in ***T****_i_*_,*t*_ are involved, as only the last row of ***T****_i_*_,*t*_ is useful for the estimation of attitude. We replace the transformation matrix ***T****_i_*_,*t*_ in Equation (17) with the state vector ***X****_i_*_,*t*_, which is defined in Equation (13). The new integration can be given by

(18) $$X_{i,t}=\left(I_{3}+\Delta t{\tilde{\omega }}_{i,t-1}\right)X_{i,t-1}$$

where ***ω*** can only be measured by the gyroscope in the IMU, which is modeled in Equation (9a). We substitute Equation (9a) into Equation (18) to obtain

(19) $$X_{i,t}=\left(I_{3}+\Delta t{\tilde{y}}_{i,G,\;\;t-1}\right)X_{i,t-1}+\Delta t{\tilde{X}}_{i,t-1}n_{i,G}.$$

Comparing Equation (19) with Equation (16a), the state transition matrix and the noise in the process can be expressed as:

(20a) $$\Phi_{i,t-1}=I_{3}+\Delta t{\tilde{y}}_{i,G,\;\;t-1}$$

(20b) $$w_{i,t-1}=\Delta t{\tilde{X}}_{i,t-1}n_{i,G}$$

When combined with Equation (15), the covariance of the noise in the process can be expressed as:

(21) $$Q_{i,t-1}=-\Delta t^{2}{\tilde{X}}_{i,t-1}E\left[n_{i,G}n_{i,G}{}^{T}\right]{\tilde{X}}_{i,t-1}$$

where *E* is the expectation operator, and the covariance of the noise from the gyroscope [27] is equal to *σ_i_*_,G_^2^***I***_3_, which is based on the covariance being the same in different directions.

In order to improve the accuracy of estimations, we identify the external acceleration with the kinematic model of the target vehicle [10]. Equations (1a)–(1c) can be used to determine the external acceleration of the front frame while neglecting some minor terms. Finally, the kinematic model can be given by:

(22a) $$a_{y1}={\dot{v}}_{y1}+\omega_{z1}v_{x1}$$

(22b) $$a_{z1}={\dot{v}}_{z1}-\omega_{y1}v_{x1}$$

where the longitudinal velocity *v_x_*_1_ is vital to the model, and the angular velocity *ω_x_*_1_ and *ω_z_*_1_ can be replaced by data from the IMU.

In addition, ${\dot{v}}_{y1}$ and ${\dot{v}}_{z1}$ can be described with a first-order low-pass filter [10]:

(23a) $$a_{1y,t}=c_{1y,a}a_{1y,t-1}+\varepsilon_{t}$$

(23b) $$a_{1z,t}=c_{1z,a}a_{1z,t-1}+\varepsilon_{t}$$

where *c*_1y,a_ and *c*_1z,a_ are constant between 0 and 1 and determine the cut-off frequency [10], which is considered to be equal to *c*_a_, and *ε_t_* is the error in this model, which is time-varying. We substitute Equations (22a) and (22b) into Equations (23a) and (23b), respectively, to obtain:

(24) $$a_{t,1}=c_{a}a_{t-1}+y_{Gt,1}v_{t}+n_{G,1}n_{v,1}+\varepsilon_{t}$$

where ***a****_t_*_,1_ = [*a*_1*y,t*_ *a*_1*z,t*_]^T^, ***y***_Gt,1_ = [*G*_1*z,t*_ *G*_1*x,t*_]^T^, ***n***_G,1_ = [*n*_Gz_ *n*_Gx_]^T^, ***n****_v_*_,1_ = [*n_v_ n_v_*]^T^, and ***ε***_t_ = [*ε*_yt_ *ε*_zt_]^T^.

Then, we substitute Equation (5b) into Equation (24) to obtain:

(25) $$y_{At,1}=H_{m}gX_{t}+c_{a}a_{t-1}+y_{Gt,1}v_{t}+n_{G,1}n_{v,1}+\varepsilon_{t}+n_{A,1}$$

where ***y****_At_*_,1_ = [*A_y,t_ A_z,t_*]^T^, *H_m_* is a matrix for coping with our disregard of the acceleration along the X-axis, and ***n****_A_*_,1_ = [*n_Ay_ n_Az_*]^T^.

In order to correspond with the measurement model in Equation (16b), Equation (25) can be written as:

(26) $$y_{At,1}-c_{a}a_{t-1}-y_{Gt,1}v_{t}=H_{m}gX_{t}+n_{G,1}n_{v,1}+\varepsilon_{t}+n_{A,1}.$$

Then, we can obtain

(27a) $$z_{t,1}=y_{At,1}-c_{a}a_{t-1}-y_{Gt,1}v_{t}$$

(27b) $$H_{1}=gH_{m}$$

(27c) $$v_{t}=n_{G,1}n_{v,1}+\varepsilon_{t}+n_{A,1}.$$

Then, we can obtain the improved estimate of ***X****_1,t_*(2) and ***X****_1,t_*(3) in the state vector ***X****_1,t_*. All the terms of the vector satisfy:

(28) $$X_{1,t}{\left(1\right)}^{2}+X_{1,t}{\left(2\right)}^{2}+X_{1,t}{\left(3\right)}^{2}=1.$$

This provides an approach to calculating ***X****_1,t_*(1) in combination with the measurement model, which can be given by [27]

(29) $$y_{Ax,t}=\sqrt{1-X_{1,t}{\left(2\right)}^{2}+X_{1,t}{\left(3\right)}^{2}}+\gamma_{t}.$$

Then, we can obtain

(30a) $$z_{t,2}=y_{Ax,t}$$

(30b) $$H_{2}=\left[\begin{array}{ccc} 1 & 0 & 0 \end{array}\right]$$

(30c) $$v_{t,2}=\gamma_{t}.$$

After the external acceleration of the front frame has been obtained, on the one hand, the data regarding acceleration from the IMU can be used to estimate the attitude of the front frame; on the other hand, these data can also be used to estimate the corresponding acceleration of the rear frame by the derived linear kinematic constraints. The attitude of the front frame can also be used to estimate the attitude of the rear frame with the angular constraints.

According to Equations (3a)–(3c), the acceleration of the rear frame can be expressed as

(31) $$y_{At,2}-o_{t}\left(a_{t,1}\right)=gX_{2,t}+n_{A,2}$$

where ***y****_At_*_,2_ = [*A_x,t_ A_y,t_ A_z,t_*]^T^ and *o_t_* (***a***_t,1_) = [*A_x,t_ A_y,t_ A_z,t_*]^T^.

Then, we can obtain

(32a) $$z_{t,3}=y_{At,2}-o_{t}\left(a_{t,1}\right)$$

(32b) $$H_{3}=gI_{3}$$

(32c) $$v_{t,3}=n_{A,2}.$$

Similarly, the pitch and roll of the rear frame can be obtained with Equations (12a) and (12b).

According to Equations (3a)–(c), the attitude vector of the rear frame can also be expressed as a function of the front frame:

(33) $$\left[\begin{array}{c} \theta_{2}\\ \begin{array}{c} \phi_{2}\\ \psi_{2} \end{array} \end{array}\right]=\left[\begin{array}{ccc} \cos\varphi & \sin\varphi & 0\\ \sin\varphi & \cos\varphi & 0\\ 0 & 0 & -1 \end{array}\right]\left[\begin{array}{c} \theta_{1}\\ \begin{array}{c} \phi_{1}\\ \psi_{1} \end{array} \end{array}\right]+\left[\begin{array}{c} 0\\ 0\\ 1 \end{array}\right]\varphi .$$

In a general way, we can obtain the attitude of the rear frame by substituting the estimation of the attitude of the front frame into Equation (33).

#### 2.3.2. KF for Yaw

According to Equations (7), (12a) and (12b), the KF for yaw can be expressed as

(34a) $$X_{z,t}=A_{z,t-1}X_{z,t-1}+Bu_{z,t-1}+w_{z,t-1}$$

(34b) $$z_{z,t}=H_{z}X_{z,t}+v_{z,t}$$

where $A_{z,k-1}=k_{1}L_{f1}\left(L_{f2}-L_{f1}\right)/\left(\left(I_{z1}+m_{1}L_{f2}^{2}\right)v_{x,}{}_{k-1}\right)$, $B_{z,k}=\left[\begin{array}{cc} 1/\left(I_{z1}+m_{1}L_{f2}^{2}\right) & -k_{1}\left(L_{f2}-L_{f1}\right)/\left(I_{z1}+m_{1}L_{f2}^{2}\right) \end{array}\right]$, and $u_{z,k}={\left[\begin{array}{cc} T_{0,k} & \beta_{k} \end{array}\right]}^{T}$, $H_{z}=1$.

Then, the yaw of the front frame can be obtained by:

(35) $$\left\{\begin{array}{l} {\hat{X}}_{z,k}^{-}=A_{z}{\hat{X}}_{z,k-1}+B_{z}u_{z,k-1}\\ P_{z,k}^{-}=A_{z}P_{z,k-1}A_{z}^{T}+Q_{z}\\ K_{z,k}=P_{z,k}^{-}H_{z}{}^{T}{\left(H_{z}P_{z,k}^{-}H_{z}{}^{T}+R_{z}\right)}^{-1}\\ {\hat{X}}_{z,k}={\hat{X}}_{z,k}^{-}+K_{z,k}\left(z_{z,k}-H_{z}{\hat{X}}_{z,k}^{-}\right)\\ P_{z,k}=\left(I-K_{z,k}H_{z}\right)P_{z,k}^{-}{\left(I-K_{z,k}H_{z}\right)}^{T}+K_{z,k}R_{z}K_{z,k}{}^{T} \end{array}\right..$$

The corresponding part of the rear frame can be obtained with Equation (3c).

### 2.4. Fusion of States

The attitude of the rear frame, which can be obtained by the linear and angular constraints, should be fused to improve the accuracy of estimations. The attitudes from different sources can be expressed as:

(36a) $${\bar{X}}_{t}={\left[\begin{array}{cc} {\bar{\theta }}_{2,t} & {\bar{\phi }}_{2,t} \end{array}\right]}^{T}$$

(36b) $${\overset{⌢}{X}}_{t}={\left[\begin{array}{cc} {\overset{⌢}{\theta }}_{2,t} & {\overset{⌢}{\phi }}_{2,t} \end{array}\right]}^{T}$$

where ${\bar{X}}_{t}$ and ${\overset{⌢}{X}}_{t}$ are the estimated value from the angular and linear constraints, respectively.

The fusion of the data can be given by

(37) $${\hat{X}}_{2,t}=\left[U_{1,t}^{-1}+U_{2,t}^{-1}\right]\left(U_{1,t}^{-1}{\bar{X}}_{t}+U_{2,t}^{-1}{\overset{⌢}{X}}_{t}\right)$$

where $U_{1,t}^{-1}$ and $U_{2,t}^{-1}$ are the corresponding estimation errors, respectively.

## 3. Simulation Verification

To verify the validity and accuracy of the proposed method, a simulation was performed with a verified prototype model of the LHD vehicle in ADAMS, and the relevant parameters were shown in Appendix A. The data were processed with the proposed method to estimate the attitude of the LHD vehicle.

### 3.1. Simulation Setup

According to [15,25], the sampling frequency of the simulation was set to 100 Hz. The speed of the LHD vehicle was set to 14 km/h under normal running conditions and 9.5 km/h during the constant radius steering process. The noise variances of the accelerometer and the gyroscope were set to 0.4 mg/$\sqrt{Hz}$ and 0.02 dps/$\sqrt{Hz}$, respectively. The noise variances of the encoder and the velocity were set to 0.01 dps/$\sqrt{Hz}$ and 0.2 m/s/$\sqrt{Hz}$, respectively.

### 3.2. Simulation Conditions

**Figure 5:**
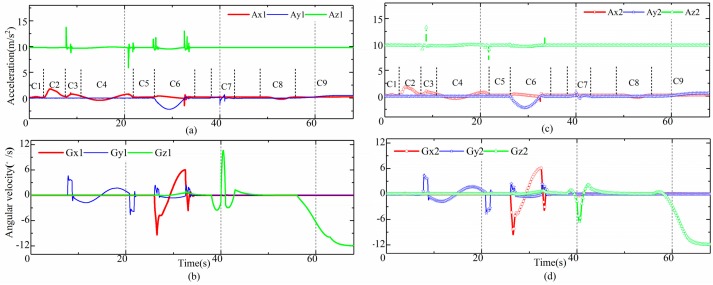
Data from the inertial measurement units (IMUs). (**a**) acceleration data of front frame; (**b**) angular velocity data of front frame; (**c**) acceleration data of rear frame; (**d**) angular velocity data of rear frame.

**Figure 6:**
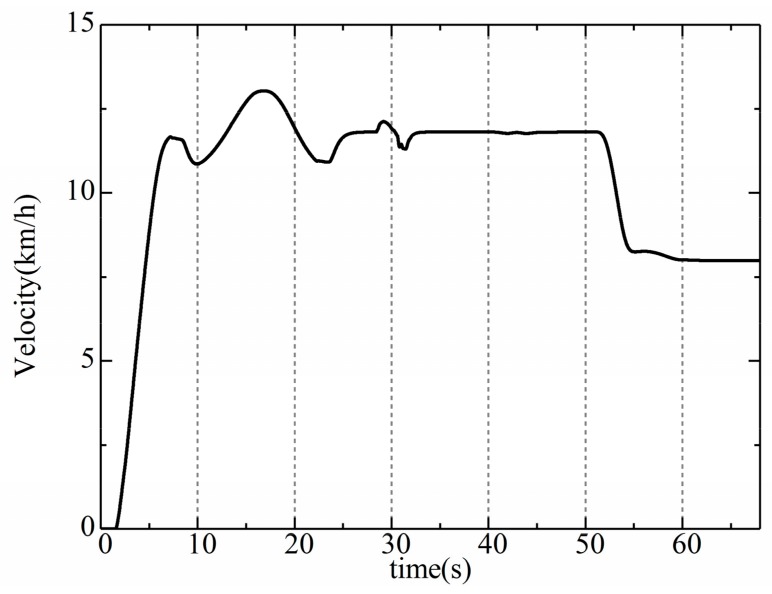
The velocity of the LHD vehicle.

In order to test the performance of the proposed method thoroughly, we included nine conditions, which are the static condition (C1, 0–3 s), the acceleration condition (C2, 3–8 s), the obstacle condition (C3, 9–10 s), the sine condition (C4, 10–20 s), the steady-state condition (C5, 22–25 s), the unilateral bridge condition (C6, 26–34 s), the single lane change condition (C7, 38–42 s), the break condition (C8, 45–48 s), and the constant radius steering condition (C9, 55–68 s). The acceleration data and the angular velocity data from the IMUs are shown in Figure 5 and Figure 6, respectively.

As we can see from Figure 5, during C1, the LHD vehicle was static, the accelerations in the lateral and longitudinal directions were zero, and the accelerations of two of the frames in the vertical direction were the same as gravity. During C2, the LHD vehicle accelerated in a longitudinal direction according to the acceleration data in Figure 5a, and the corresponding angular velocity was very small because the LHD vehicle has no suspension. During C3, the left and right front wheels of the LHD vehicle simultaneously encountered a bump; as a result, the vertical accelerations in vertical motion and pitch motion changed dramatically. During C4, the LHD vehicle ran over a sine bump, and the pitch motion changed with the rise and fall of the road. After that, the LHD vehicle ran at a constant speed, C5, which was similar to the speed during C1.

According to Figure 6, the velocity of the LHD vehicle oscillated during C4 and C5. This was caused by the rough control strategy for velocity during the simulation. However, the range remained within the LHD vehicle’s normal running velocity range.

Then, the left wheel of the LHD vehicle encountered a sine bump, C6, which brought about changes in the roll motion, and the lateral acceleration also changed with the rise and fall of the road. During C7, the yaw motion of the front frame and the rear frame changed obviously. During C8, the acceleration in the longitudinal direction, in contrast to C2, was negative, and the speed of the LHD vehicle was also reduced to 9.5 km/h. Then, the LHD vehicle steered in a constant radius. At the start of C9, the angular velocities of the front frame and the rear frame were different, which implies that the angles of central articulation had changed.

### 3.3. Estimation Results

**Figure 7:**
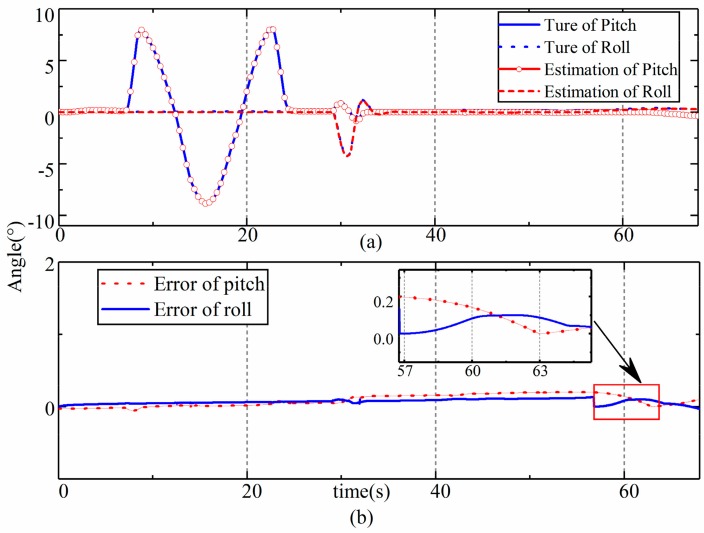
Results of the estimation of pitch and roll. (**a**) estimation results of pitch and roll; (**b**) error of pitch and roll estimation.

**Figure 8:**
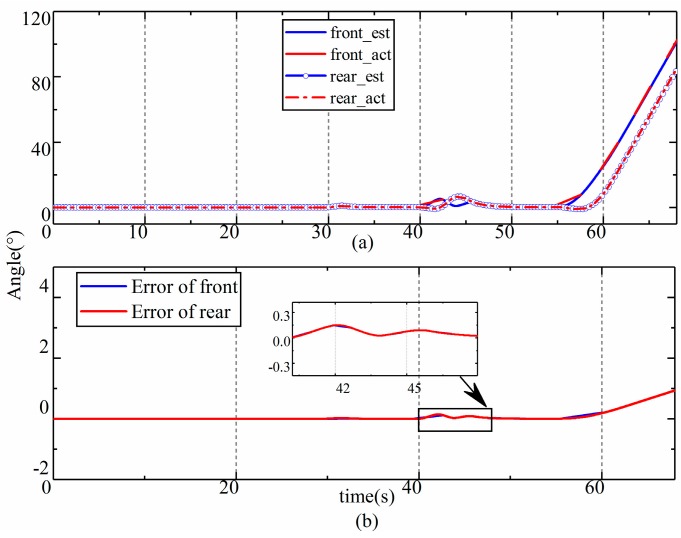
Error in the estimation results. (**a**) estimation results of yaw; (**b**) error of yaw estimation.

**Figure 9:**
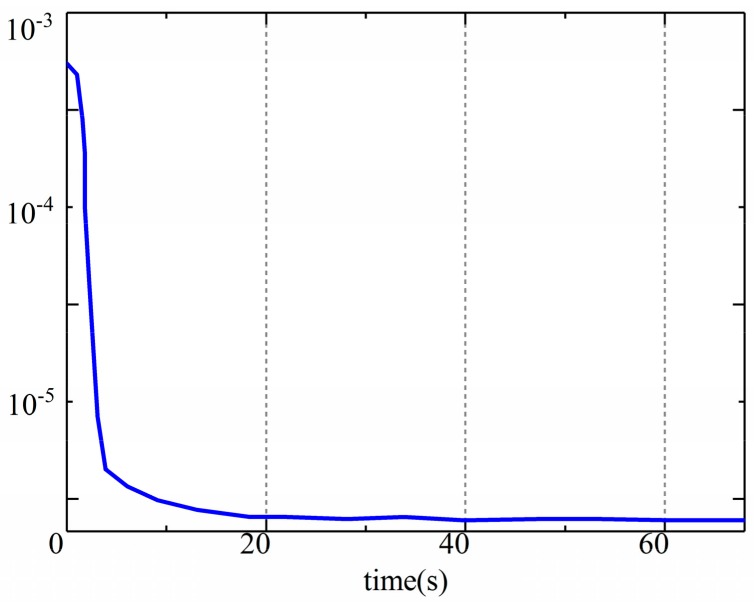
Trace of error covariance matrix.

The results of the estimation of the pitch, roll, and yaw angles of the LHD vehicle are shown in Figure 7 and Figure 8, respectively, and the time history of the error covariance matrix during the estimation was shown in Figure 9. The upper part in the figures is a comparison between the estimated values and the true values, and the lower part shows the errors in the estimation. We found that the roll and pitch of the front frame and the rear frame were almost the same, so only the attitude of the rear frame is shown in Figure 7; however, we included the yaw of the two frames. As we can see from Figure 7, Figure 8 and Figure 9, the estimated values and the true values are in good agreement. The RMS of errors in the roll, pitch, and yaw were 0.2° (2.4%), 0.19° (2.1%), and (1.1°) 0.23%, respectively. The percentage was obtained with the maximum RMS and the largest values of corresponding angles. The maximum error in the estimations appeared in C9 (the constant radius steering condition), which was caused by the slip of the LHD vehicle during steering. Although small differences appeared during the dynamic process according to the error, the estimations were found to be satisfactory.

**Figure 10:**
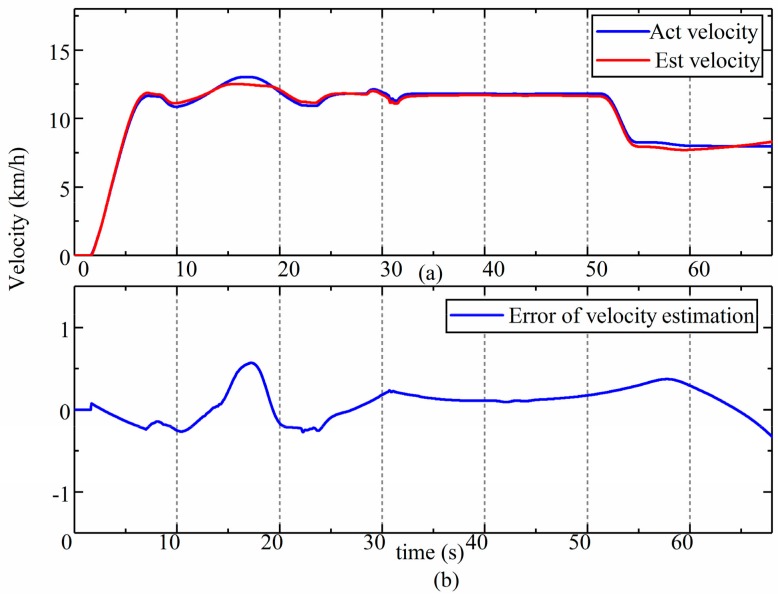
Results of the estimation of velocity. (**a**) estimation results of velocity; (**b**) error of velocity estimation.

Then, the estimated longitudinal accelerations of the rear frame were integrated to obtain the velocity of the LHD vehicle. Figure 10a compares the estimated velocity with the reference velocity. Figure 10b shows the error in the estimation of the velocity. During 15–18 s condition, the drastic changes of the pitch angle and characteristic of the tire would increase the estimation error of acceleration and the dynamic model, and the error of acceleration caused the deviation of velocity. Therefore, a deviation occurred. The error in the estimated speed was less than 0.27 km/h (3%). This means that the proposed method can be used in the control and navigation of an LHD vehicle without any infrastructure.

## 4. Experimental Results

### 4.1. Experimental Setup

**Figure 11:**
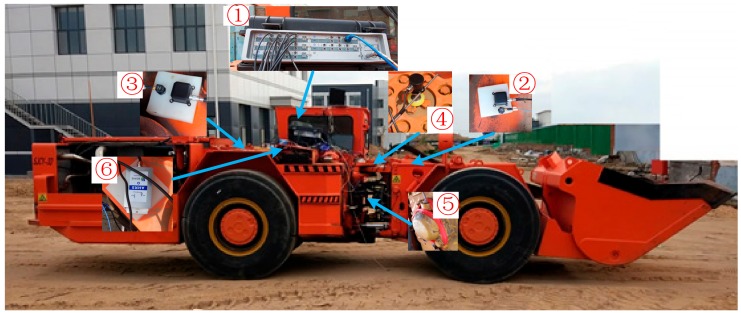
The system configuration of the LHD vehicle field test.

The field test of the LHD vehicle was carried out on an uneven road that was similar to the road in underground mines. The test system is shown in Figure 11. The LMS SCADAS (①, SCM05) was used to record the data during the field test, and the sample frequency was set to 100 Hz according to [15]. Two IMUs (②, ③, Beijingsanchi, SC-AHRS-200A) were installed in the front frame and the rear frame, respectively. Two kinds of encoders were used to measure the articulated angle of a frame (④, MIRAN, WOA-C) and the speed of transmission (⑤, SCHMEASAL IFL 5-18M-10P), respectively. A gyroscope (⑥) with high precision (the bias stability is less than 0.05 °/h, and the random walk coefficient is less than 0.005 °/$\sqrt{Hz}$) was installed to provide references.

**Figure 12:**
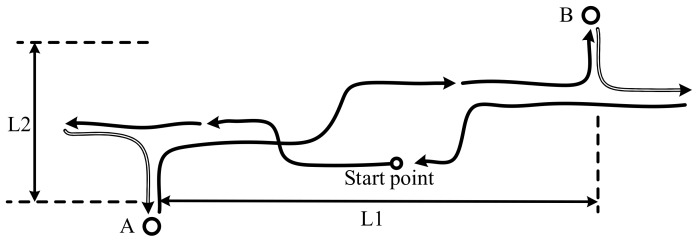
The route of the LHD vehicle during the field test.

Different conditions were adopted to simulate the working conditions of the LHD vehicle. Usually, the characteristics of the route of an LHD vehicle are special in underground mines, and always include a square turn and a change in operation direction. Thus, the route for the field test was planned as shown in Figure 12, where the direction of the arrow represents the direction of the LHD vehicle and the solid and hollow lines correspond to the forward and backward directions, respectively. The key dimensions are L1 = 50 m and L2 = 20 m. The operations between Point A and Point B were used to simulate the operations between the blast point and the dump point of the underground mines [2]. Although the trajectory in the field test was not so long as the other autonomous ground vehicle, the operation and dynamic characteristics in this route can cover all of characteristics of the LHD during the normal operations.

### 4.2. Experimental Results

**Figure 13:**
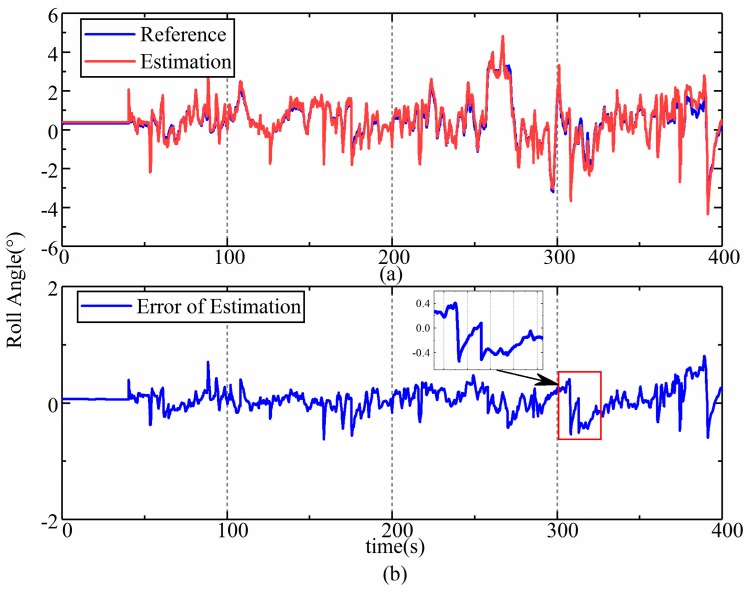
Results of the estimation of roll during the field test. (**a**) estimation results of roll in field test; (**b**) error of roll estimation in field test.

**Figure 14:**
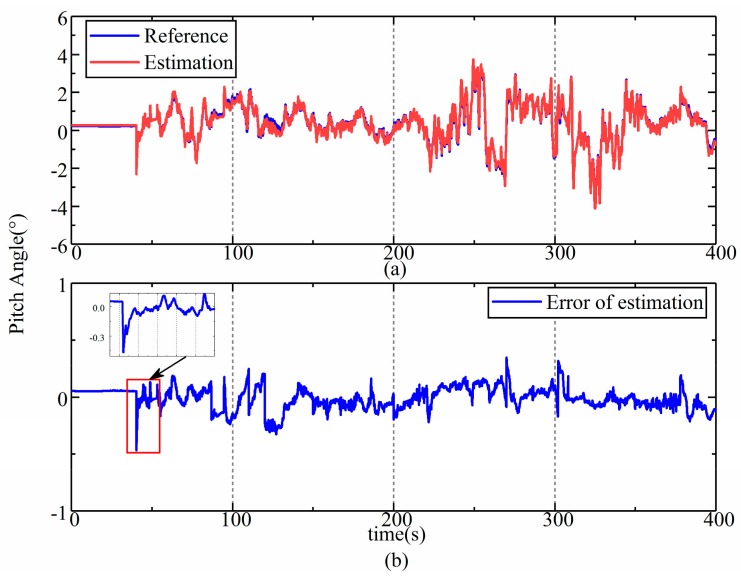
Results of the estimation of pitch during the field test. (**a**) estimation results of pitch in field test; (**b**) error of pitch estimation in field test.

**Figure 15:**
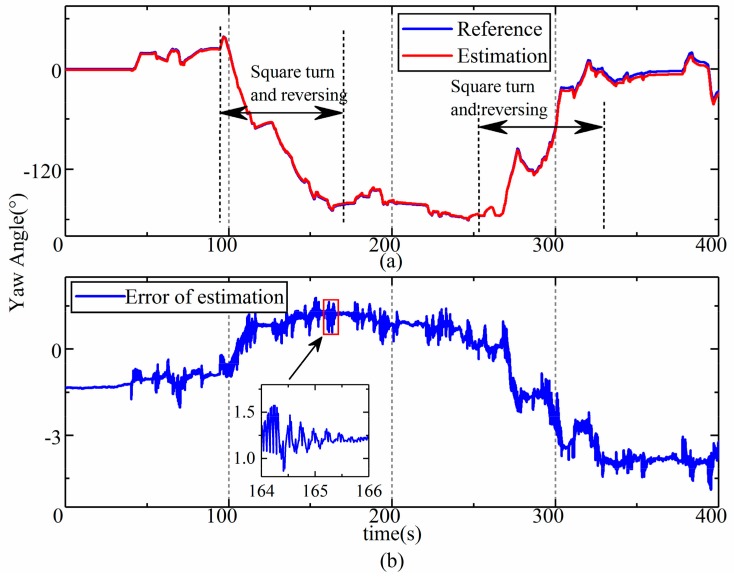
Results of the estimation of yaw during the field test. (**a**) estimation results of yaw in field test; (**b**) error of yaw estimation in field test.

The results of the estimation of the front and rear frames were almost the same according to the data from the field test; thus, only the results of the estimation of the rear frame’s attitude angle are shown and were analyzed. The results of the estimation corresponding to the roll, pitch, and yaw are shown in Figure 13, Figure 14 and Figure 15, respectively. The upper part (a) in each figure is a comparison between the reference values and the estimation, and the lower part (b) shows the error in the estimation, and the dramatic or typical changes of errors were detailed. The reference values were sourced from the data of a high-precision reference gyroscope.

### 4.3. Discussion of Results

As we can see from Figure 13 and Figure 14, the estimations of roll and pitch are consistent with the reference values, while some errors fluctuate sharply. This is due to the special structure of the LHD vehicle’s chassis, which is also a common characteristic of UAVs. The frames of an LHD vehicle are rigidly connected to the front and rear drive axle, which means that these vehicles do not have suspension that can filter vibrations from the road. Although rubber pads are used to eliminate sharp fluctuations, the improvement is limited. Hence, vibrations bring about sharp fluctuations in the errors in the estimation.

**Figure 16:**
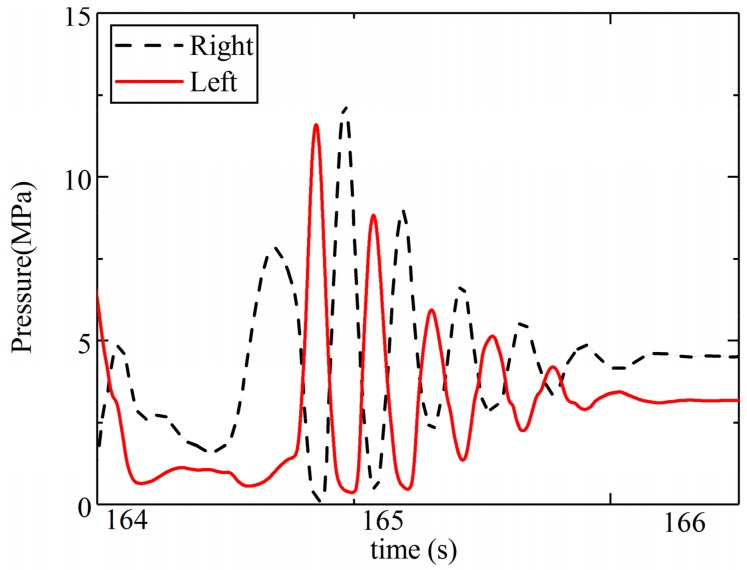
The pressure in the steering cylinders.

As we can see from Figure 15, the estimation of yaw is highly consistent with the reference value, and the error is less than 5 degrees and does not increase with time. The error only obviously changed after the square turn (100–150 s, 260–310 s). During the square turn, the tire of the LHD vehicle seriously slipped, so error would be brought about by the change in Equations (8a) and (8b). In addition, according to Figure 16, ripples also occurred between 164 s and 166 s. This error could be traced back to the input of the estimation model, which is the steering moment. The steering moment is decided by the pressure in the cylinder. The pressure is shown in Figure 16, where “right” and “left” refer to the rod-less cavity pressure in the right and left steering cylinder, respectively. As we can see from the Figure 16, the two pressures rippled alternately—between 12.5 MPa and 0.2 MPa—after a steering maneuver. This means that the oscillatory yaw motion in the frames occurred, which is always caused by instability in the hydraulic steering system. Although different errors occurred during the estimation, the accuracy was found to meet the requirements of practical applications.

**Figure 17:**
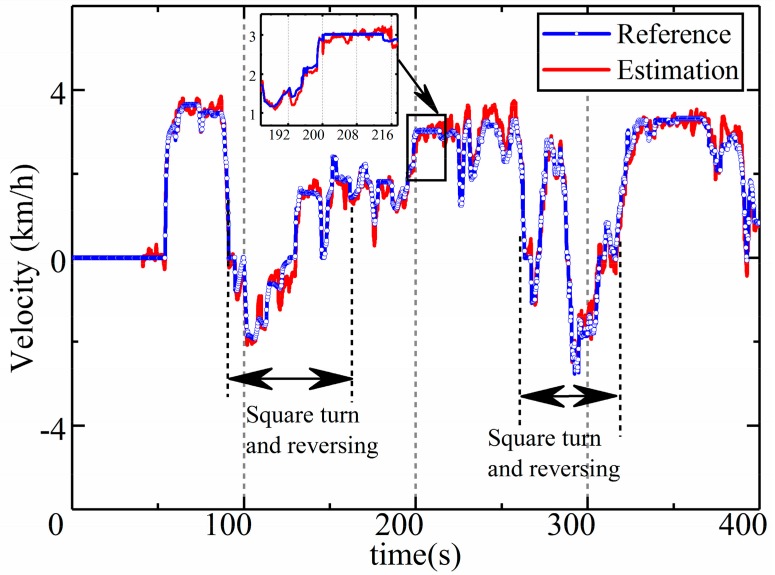
Estimation of velocity during the field test.

During the estimation, the estimated value of the longitudinal dynamic acceleration is converted to the velocity of the LHD vehicle by integration. Figure 17 compares the estimated velocity obtained from the shaft encoder with the reference value. As we can see from the figure, not only are the trends consistent, but the estimated and reference values are also very close according to the details in Figure 17.

Despite all this, the accuracy of the estimation is high enough to indicate the attitude and speed of articulated vehicles, which can be verified by the root mean square (RMS) of the error in the estimated values, which are listed in Table 1. The RMS of errors for the roll, pitch, and yaw were 0.19° (3.8%), 0.1° (2.4%), and 2.08° (4.2%), respectively. The error in the estimated velocity was 0.21 km/h (1.2%). Compared with the estimation error of 5% in [9], the proposed model-based method improves the accuracy of estimations and involves more information on attitude, which is useful for the navigation and control of autonomous vehicles [28].

## 5. Conclusions

A model-based method was presented for estimating the attitude of articulated vehicles in a GPS-free environment. A kinematic model, a two-constraint model of articulation, and a dynamic four degrees-of-freedom (DOF) yaw model of UAVs were established and integrated with rough data from IMUs by KFs to obtain information on attitude. Then, the method was verified by a simulation under different dynamic conditions. A field test of the LHD vehicle was carried out to prove the method’s practical efficacy. 

The results of the field test showed that the method could estimate the yaw and velocity accurately. The errors in the estimated roll, pitch, and yaw were 3.8%, 2.4%, and 4.2%, respectively. The error in the estimated velocity was 1.2%. The characteristics of these errors were analyzed and interpreted. The errors in the estimated pitch and roll were brought about by the direct vibrations from the ground. The errors in the estimated yaw included trend errors and ripples. The trend errors occurred at the square turn, when the constraints between the tires and the road changed. Local ripples were induced by instability in the hydraulic steering system.

A trajectory of the typical work cycle for LHD was designed during the validation field test. The field test can prove the validation of this method during the work cycle, which is most of working conditions of the LHD vehicle. For the rarely long-traveled transition, this method still needs further verification.

Therefore, motivated by these limitations and sources of error, future research will focus on improvements in accuracy. The method would be verified during the long-traveled transition, the dynamic yaw model will be improved by covering tire slips, and instability in the hydraulic steering system will be studied and controlled to eliminate ripples. In addition, the algorithm will also be improved to decrease the effect that vibrations have on the frame.

## Figures and Tables

**Table 1:** Root mean square (RMS) error in the estimation.

Error	RMS
Roll	0.19 deg
Pitch	0.10 deg
Yaw	2.08 deg
Velocity	0.21 km/h
Distance	0.26 m

## Appendix A

**Table A1:** Parameters of the target LHD.

Symbol	Parameters	Values
m_1_	mass of front frame	12,850 kg
m_2_	mass of rear frame	15,650 kg
Iz1	moment inertia of front frame	307,420 kg·m^2^
Iz2	moment inertia of rear frame	380,320 kg·m^2^
L_f1_	distance of front mass center to bridge	0.3 m
L_f2_	distance of front mass center to articulation point	2.05 m
L_r1_	distance of rear mass center to bridge	0.16 m
L_r2_	distance of rear mass center to articulation point	1.59 m
B	half distance of track	0.89 m
R	free radius of tire	0.89 m
*f_v_*	vertical stiffness of tire	80 KN/m

## References

[1] Li J., Zhan K. (2018). Intelligent Mining Technology for an Underground Metal Mine Based on Unmanned Equipment. Engineering.

[2] Gustafson A., Lipsett M., Schunnesson H., Galar D., Kumar U. (2014). Development of a Markov model for production performance optimisation. Application for semi-automatic and manual LHD machines in underground mines. Int. J. Min. Reclam. Environ..

[3] Gao Y., Shen Y., Xu T., Zhang W., Güvenç L. (2018). Oscillatory Yaw Motion Control for Hydraulic Power Steering Articulated Vehicles Considering the Influence of Varying Bulk Modulus. IEEE Trans. Control Syst. Technol..

[4] Nayl T., Nikolakopoulos G., Gustafsson T. (2015). Effect of kinematic parameters on MPC based on-line motion planning for an articulated vehicle. Robot. Auton. Syst..

[5] Yang L., Yue M., Ma T. (2019). Path Following Predictive Control for Autonomous Vehicles Subject to Uncertain Tire-ground Adhesion and Varied Road Curvature. Int. J. Control. Syst..

[6] Naisi Z., Jun N., Jibin H. (2019). Robust *H*_∞_ state feedback control for handling stability of intelligent vehicles on a novel all-wheel independent steering mode. IET Intell. Transp. Syst..

[7] Gao S., Liu Y., Wang J., Deng W., Oh H. (2016). The Joint Adaptive Kalman Filter (JAKF) for Vehicle Motion State Estimation. Sensors.

[8] Roberts J.M., Duff E.S., Corke P.I. (2002). Reactive navigation and opportunistic localization for autonomous underground mining vehicles. Inf. Sci..

[9] Chun J.T.L.Y. (2015). State Estimation of the Electric Drive Articulated Dump Truck Based on UKF. J. Harbin Inst. Technol. (New Ser.).

[10] Lee J.K., Park E.J., Robinovitch S.N. (2012). Estimation of Attitude and External Acceleration Using Inertial Sensor Measurement during Various Dynamic Conditions. IEEE Trans Instrum. Meas..

[11] Shi G., Li X., Jiang Z. (2018). An Improved Yaw Estimation Algorithm for Land Vehicles Using MARG Sensors. Sensors.

[12] Lee C.J., Kim K.E., Lim M.T. (2018). Sensor fusion for vehicle tracking based on the estimated probability. Iet Intell. Transp. Syst..

[13] Xu Z., Yang W., You K., Li W., Kim Y.I. (2017). Vehicle autonomous localization in local area of coal mine tunnel based on vision sensors and ultrasonic sensors. PLoS ONE.

[14] Dissanayake G., Sukkarieh S., Nebot E., Durrant-Whyte H. (2001). The aiding of a low-cost strapdown inertial measurement unit using vehicle model constraints for land vehicle applications. IEEE Trans. Robot. Autom..

[15] Mäkelä H. Navigation System for LHD Machines. Proceedings of the IFAC Intelligent Autonomous Vehicles.

[16] Chi H., Zhan K., Shi B. (2012). Automatic guidance of underground mining vehicles using laser sensors. Tunn. Undergr. Space Technol..

[17] Mäkelä H. (2001). Overview of LHD navigation without artificial beacons. Robot. Auton. Syst..

[18] Scheding S., Dissanayake G., Nebot E.M. (2001). An experiment in autonomous navigation of an underground mining vehicle. IEEE Trans. Robot. Autom..

[19] Paraszczak J., Gustafson A., Schunnesson H. (2015). Technical and operational aspects of autonomous LHD application in metal mines. Int. J. Min. Reclam. Environ..

[20] Gustafson A., Schunnesson H., Kumar U. (2015). Reliability Analysis and Comparison between Automatic and Manual Load Haul Dump Machines. Qual. Reliab. Eng. Int..

[21] Marshall J., Barfoot T., Larsson J. (2008). Autonomous underground tramming for center-articulated vehicles. J. Field Robot..

[22] Wu D., Meng Y., Gu Q., Ma F., Zhan K. (2017). A novel method for estimating the heading angle for underground Load-Haul-Dump based on Ultra Wideband. Trans. Inst. Meas. Control.

[23] Park B., Myung H. (2014). Underground localization using dual magnetic field sequence measurement and pose graph SLAM for directional drilling. Meas. Sci. Technol..

[24] Zhu Q., Xiao C., Hu H., Liu Y., Wu J. (2018). Multi-Sensor Based Online Attitude Estimation and Stability Measurement of Articulated Heavy Vehicles. Sensors.

[25] Sabatini A.M. (2006). Quaternion-based extended Kalman filter for determining orientation by inertial and magnetic sensing. IEEE Trans. Bio-Med. Eng..

[26] Suh Y.S. (2010). Orientation Estimation Using a Quaternion-Based Indirect Kalman Filter With Adaptive Estimation of External Acceleration. IEEE Trans. Instrum. Meas..

[27] Oh J.J., Choi S.B. (2012). Vehicle Velocity Observer Design Using 6-D IMU and Multiple-Observer Approach. IEEE Trans. Intell. Transp. Syst..

[28] Ahmed H., Tahir M. (2017). Accurate Attitude Estimation of a Moving Land Vehicle Using Low-Cost MEMS IMU Sensors. IEEE Trans. Intell. Transp. Syst..

[29] Eltrass A., Khalil M. (2018). Automotive radar system for multiple-vehicle detection and tracking in urban environments. IET Intell. Transp. Syst..

[30] He Y., Khajepour A., McPhee J., Wang X. (2005). Dynamic modelling and stability analysis of articulated frame steer vehicles. Int. J. Heavy Veh. Syst..

